# Evidence for a metacognitive awareness of autobiographical memory organisation

**DOI:** 10.1038/s41598-023-34389-0

**Published:** 2023-09-20

**Authors:** Fabien Carreras, Chris J. A. Moulin

**Affiliations:** 1 University Grenoble Alpes, University Savoie Mont Blanc, CNRS, LPNC, 38000 Grenoble, France; 2 Swansea University, SA2 8PP Swansea, Wales

**Keywords:** Psychology, Human behaviour

## Abstract

Models of autobiographical memory (AM) recall posit some form of control process, but the extent to which we can reflect on this form of retrieval is under-researched. Here we propose a method for measuring such metacognitive awareness in AM. Since the verification of personal facts is difficult, we based our design on AM organisation. AMs are proposed to be organised into a coherent life story, that is, a subjective chronology reflecting the goals of the individual over time. We investigated the metacognitive awareness of this coherence. Eighty-three participants generated AMs and made two judgements of order for pairs of memories and gave a confidence rating. We found that participants were indeed able to distinguish pairs of memories that were coherent with their life story chronology from pairs which were not. We also found a significant effect of response time and task difficulty on confidence, suggesting that judgement of order fluency was determinant for metacognitive evaluation. This suggests common properties between metacognitive abilities related to autobiographical memory and those related to other forms of memory.

## Introduction

Autobiographical memory (AM) and metacognition are two important research fields in psychology, and yet their intersection has been surprisingly overlooked by scientists, despite theoretical statements which point to a role for metacognition in AM retrieval. Metacognition is the ability to control and evaluate our cognitive processes^1^. AM models assume that metacognitive control processes are deployed during AM retrieval^2–7^. Such processes determine whether what is retrieved is an AM or not, or whether what is retrieved can be believed. These processes also act to generate suitable cues to prompt recall of the personal past (see Ref.^8^). This is carried out by examining memory plausibility, veracity, and correspondence to the current individual’s goals. Also, metacognitive beliefs, that is, the beliefs individuals have about their memory can influence this control^9, 10^. When control processes are satisfied, the memory reaches consciousness and when they are not, a new retrieval is undertaken (e.g. Ref.^6^). Some accounts of autobiographical retrieval propose that memory experiences and feelings metacognitively guide the construction of past events from the elaboration of a cue (e.g. Ref.^8^).

An important issue in metacognition is the difference between control and monitoring processes (see Ref.^1^). They describe a bidirectional flow of information from an object level to a meta-level which operates to modify ongoing cognitive processes in the object level (i.e. metacognitive control). This is contrasted with the evaluation of ongoing processes in line with goals and expectations (i.e. metacognitive monitoring). According to such a view, the control processes proposed to exist in autobiographical memory are not possible without appropriate monitoring: we withhold retrieving a memory (control) because we feel uncertain that it is a true representation of the personal past (monitoring). In this paper, our focus is on metacognitive monitoring.

Even though such associations between AM and metacognition have been implied, the extent to which we can readily monitor what we retrieve from AM has not been documented. A recent study by Uzer et al.^11^ using the *retrieve-aloud procedure* demonstrated that participants were able to describe what happens during AM retrieval precisely enough to allow neutral judges to predict what type of retrieval (i.e. direct or generative, see Ref.^12–15^) they were operating. Using a similar procedure, Mace et al.^16^ have recently advanced a multi-process retrieval theory of autobiographical memory, which describes processes which are in keeping with our metacognitive view of autobiographical retrieval. The two tenets of this theory are that AM retrieval relies on multiple processes and cues, and that there is ‘retrieval selectivity’ which is the capacity to adapt retrieval processes in function with retrieval goals and the nature of what has been retrieved or not. This conceptualisation of ’selectivity’ to us implies that participants should be able to monitor what is being retrieved. The aim of this project was to fill a gap in the literature. Specifically, it aimed to investigate whether individuals were able to judge the veracity of what they retrieved from their AM, that is, metacognitive monitoring related to AM.

Metacognition research distinguishes two types of task performance^17^. The first order performance is the individual performance on the task (e.g. the percentage of correct answers). The second order performance considers how accurately someone evaluates their first order performance, i.e. metacognitive monitoring (e.g. the percentage of correct answers estimated by the participant). A typical way to assess the second order performance in a memory task is to investigate the confidence participants have in their right and wrong answers. Appropriate metacognitive monitoring is shown when participants distinguish their right and their wrong answers with their confidence judgements. The focus in the present experiment is on whether subjective confidence judgements are appropriate. Usually, such an analysis considers confidence for objectively correct and incorrect responses. As we will develop below, because such objective verification is difficult in autobiographical memory, our focus is on whether participants’ confidence judgements can discriminate between responses with a coherent chronology from an incoherent chronology.

Several researchers have considered the basis for such second order performance^18^. Confidence can be influenced by several characteristics, notably first order fluency. Fluency is the subjective experience of ease or difficulty with which we are able to process information^19^. It is often measured using response time in experimental contexts. A common finding is that the more an answer is fluent, the higher the confidence^20^. Fluency is in turn influenced by task difficulty, that is, the more the task is difficult, the more the feeling of difficulty is strong, the more confidence is low^21^.

In metacognitive studies, participants’ first order performance is assessed with reference to an objectively correct answer (e.g. in a lexical decision task or word learning task; see Ref.^22^). As such, the confidence participants gave to correct and incorrect answers can be compared. AM studies encounter an issue here: AM is personal and subjective, thus researchers cannot easily judge the veracity of answers in an AM task—and it is not usual to classify autobiographical retrievals as ‘right’ or ‘wrong’ even though some aspects of accuracy and specificity are typically measured. Our view of AM is in line with Scoboria et al.’s^4^ proposal, that is, “Even in cases where photographs or diaries exist to corroborate past events, memories remain subjective appraisals that are based on the information available at the time of remembering” (p. 337–338).

Our solution was to consider the subjective organisation of memories, and have people give confidence judgements for ordering of events from their personal past in a laboratory task. Note that, if someone indicates that their wedding occurred before their graduation, we cannot be sure of the true order of these events, but we can assume that they should be coherently and consistently reported as occurring in the same order. Moreover, in line with the notion of AM being highly reconstructive^23^, research has shown that AM chronology does not perfectly match with real life chronology. For example, Burt et al.^24^ asked participants to keep a personal diary for a few months. Participants had to record events and their date of occurrence. After a delay, they had to indicate the chronology of the events they wrote in the diary and interestingly – given the aim of this paper – to indicate the confidence they had in their answers. Results showed that the chronology given by the participants differed from the diary chronology. Burt et al. found no relationship between confidence and performance in this ordering task, when gauged objectively—i.e. compared to the diary.

We argue that to better evaluate metacognition in AM, we should consider its relationship with subjective organisation, hence our focus on coherence. Indeed, AM does not necessarily correspond to reality, but is organised in order to support the individual’s goals^25^. Here we are interested in assessing AM subjective chronology. Thus, we focused on AM organisation at the time of the experiment, without trying to compare it with the objective chronology of the generated memories. In order to do this, we asked twice the order in which events occurred, and for the purposes of evaluating metacognitive accuracy suppose that the consistent responses are the ‘correct’ answers. This gives us an evaluation of the first order performance taking in consideration AM subjective organisation, and hence the possibility to assess second order performance. We acknowledge that consistent answers can be based on an incorrect objective chronology -e.g. a participant can indicate twice that their friend’s wedding occurred before theirs (even though it did not), but we argue that consistent answers reflect a subjective AM chronology -e.g. the experient may actually remember the participant’s wedding as occurring before their friend’s wedding. Even if participants would be objectively wrong, they would be consistent, and our metacognitive measure effectively taps into whether the participant is able to make consistent judgements which are coherent with a stable representation of their personal past.

**Figure 1:**
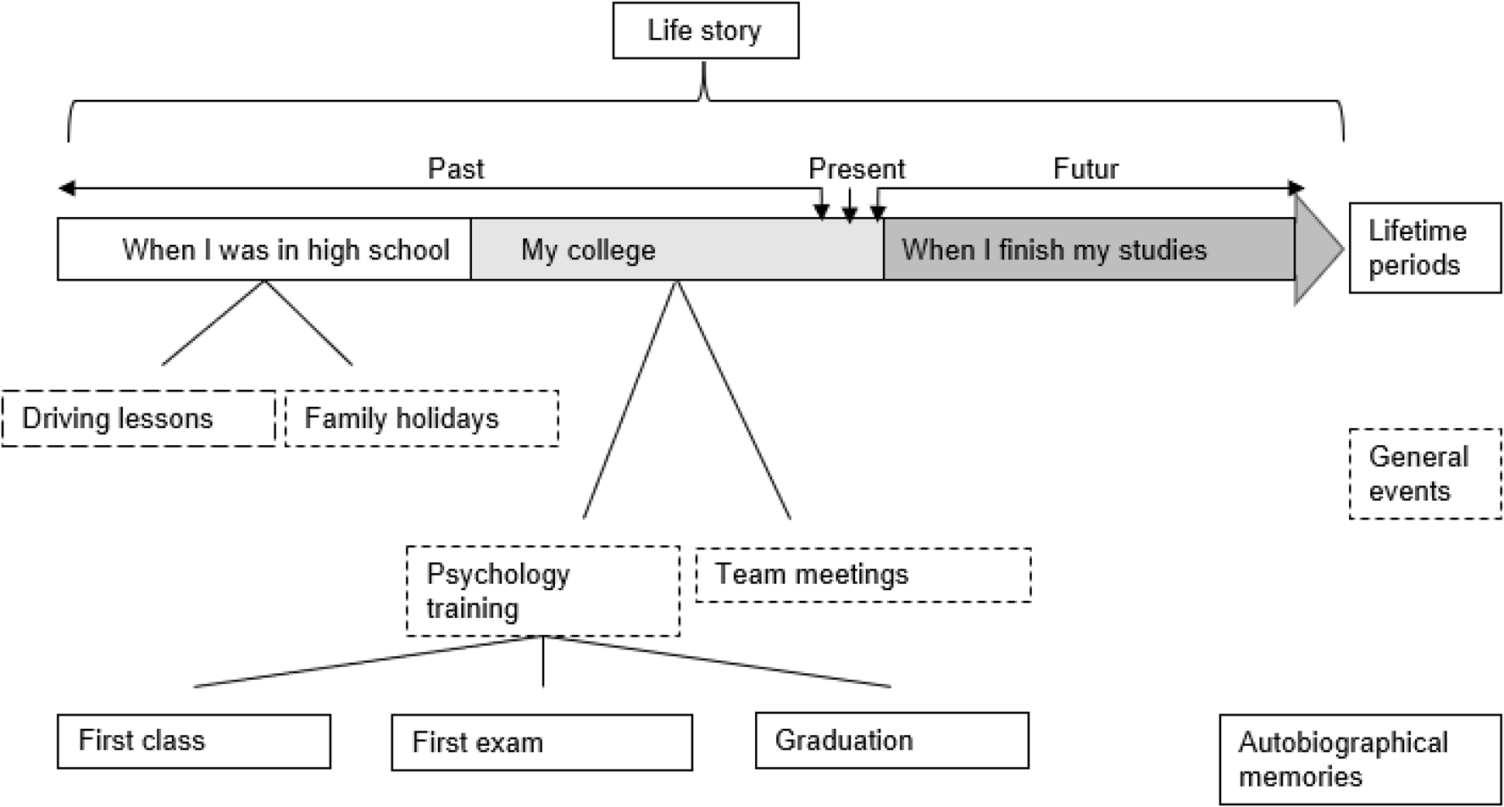
Organisation of autobiographical memory according to the self-memory system^15^.

Our task assessed autobiographical organisation according to the Self-Memory System^15^. This model postulates that AM has a hierarchical structure (Fig. 1). The highest level is the life story. It is a representation of one’s life and underlines the goals of the individual over the time^26^. The life story is divided in lifetime periods representing important periods of the individual’s life such as the different scholar periods^27^. These periods contain general events which regroup thematically or temporally close autobiographical memories. Autobiographical memories are supposed to follow a stable chronology that underlines the goals of the individual over their life^28^. This chronology can contradict the objective chronology to support the individual’s goals^25^.

Skowronski et al.^29, 30^ managed to investigate AM chronology with respect to its subjectivity. They asked participants to generate memories from two lifetime periods: their high school and their college. Generated memories were regrouped by pairs. Two types of pairs were created: Intraperiod pairs of memories, containing two memories from the same lifetime period, and interperiod pairs of memories, containing two memories from different lifetime periods. During the judgement of order task, pairs of memories were presented one at a time on a screen, and participants had to indicate either which event of the pair occurred first, or which event of the pair occurred last. Next, participants had to give the date of occurrence of every memory they generated. Each judgement of order was categorised as coherent or noncoherent with the chronology given by the dates (e.g. if the participant indicated that their graduation, 2012, occurred before their wedding, 2014, the judgement was coherent). Results showed that judgements of order were more successful when realised with interperiod pairs of memories than with intraperiod pairs of memories, that is, variations in task difficulty. Moreover, judgements of order with interperiod pairs were performed faster than judgements of order with intraperiod pairs. In this study, judgements of order were categorised as right or wrong through dates given for the events. Because dates in memory are associated with many biases and lack of precision, a judgement of order is a better way to assess AM chronology^31, 32^. Thus we proposed a new design based on multiple judgements of order.

Participants first generated autobiographical memories from two lifetime periods. These memories were organised into pairs (e.g. *my bachelor graduation day*—*The wedding of my best friend*). In a second phase, each pair of memories was subject to two judgements of order of occurrence in two separate blocks. These two judgments allowed us to distinguish two types of pairs of memories. A coherent pair of memories was a pair in which the judgements of order were consistent across blocks (e.g. if a participant indicated in one block that their graduation occurred *before* their wedding, and in the other block that their wedding occurred *after* their graduation). A noncoherent pair was a pair in which the two judgements were contradictory across blocks indicating that one judgement of order conflicted with the life story chronology (e.g. if a participant indicated in one block that their graduation occurred *before* their wedding, and in the other block that their graduation occurred after their wedding). Every judgement of order was followed by a confidence rating on a 1–7 scale with seven being the highest confidence. We thus categorised each pair as coherent or noncoherent, and hypothesised a greater confidence for coherent pairs of memories than in noncoherent pairs.

We also tested several exploratory hypotheses. We also asked participants to date their memories and we categorised every judgement of order as right or wrong the same way Skowronski et al.^30^ did. Indeed, if a judgement of order matched with date chronology, it was categorised as date-coherent judgement of order. If a judgement of order conflicted with dates chronology, it was categorised as a date noncoherent judgement of order. Next, we tried to replicate Skowronski et al.’s^29, 30^ results showing that intraperiod judgements of order (i.e. judgements of order with two memories from the same lifetime period) were less successful and slower than interperiod judgements of order (i.e. judgements of order with two memories from different lifetime periods). Finally, we explored the role of fluency in metacognitive ratings, that is, the effect of task difficulty on participants’ confidence, and the link between response time and confidence.

## Results

Our aim was to examine to what extent people can accurately ascribe confidence to their performance in an AM ordering task. There are two ways to address this question—Either more objectively, considering how participants respond to differences in the difficulty task (in this case comparing intra and inter period pairings)—And more subjectively, examining how confidence maps onto the accuracy of the first order judgement.

Our study was carried out online on the platform Pavlovia^33^. Analyses were conducted using R (version 4.1.1)^34^. Scripts, auxiliary results, and data can be found on OSF: https://osf.io/wzt2p/?view_only=544050b3f69948d089ed405932299e3f. Our main hypothesis was preregistered and tested with an alpha threshold of 0.05 (i.e. the difference of confidence between pairs of memories associated with consistent judgement of order across blocks, and pairs of memories associated with contradictory judgements of order across blocks). Other analyses were carried out after data collection and for these, we applied a Bonferonni correction and used an alpha threshold of 0.008. All *t* tests were one-tailed according to our hypotheses. When analyses revealed participants with a Standardised Deleted Residual higher than 4 (Outliers), these participants were removed from their respective analyses^35^. Participants who indicated one level of confidence through the entire experiment were excluded from analyses involving confidence. Participants without noncoherent pairs of memories (i.e. ceiling performance, n = 2) were excluded from analyses comparing coherent and noncoherent pairs of memories. Unless otherwise indicated assumption checks for realised tests were assessed using shapiro wilk tests and plots and respected. Effect sizes were calculated using Cohen’s *d*.

### Confidence analysis

There was a significant effect of coherence on confidence (Table 1). Pairs of memories associated with consistent judgements of order across blocks were associated with a greater confidence than pairs of memories associated with contradictory judgements of order, *t*(46) = 11.75, *p* < 0.001, *d* = 1.71, 90% IC [1.34, 2.11]. That is, participants were more confident in pairs in which the same chronological order was retrieved in the two judgements of order.

**Table 1:** Second order performance: mean confidence according to pair coherence and judgement of order coherence.

	Coherent (SD)	Non-coherent (SD)	Cohen’s d (90% IC)	p
Pairs of memories	6.42 (0.86)	5.08 (0.84)	1.71 ([1.34, 2.11])	< 0.001
Judgements of order	6.46 (0.84)	4.45 (1.29)	1.36 ([1.04, 1.71])	< 0.001

Standard deviations are given in brackets. A coherent pair of memories was a pair in which the judgements of order were consistent across blocks (e.g. if a participant indicated first that their graduation occurred before their wedding, and second that their wedding occurred after their graduation). A noncoherent pair was a pair in which the two judgements were contradictory (e.g. if a participant indicated first that their graduation occurred before their wedding, and second that their graduation occurred after their wedding). A judgement of order was categorised as coherent if it was in line with the chronology given by the dates (e.g. if the participant indicated that their graduation, 2012, occurred before their wedding, 2014). A judgement of order was non-coherent if it conflicted with the chronology given by the dates (e.g. if the participant indicated that their graduation, 2012, occurred after their wedding, 2014). Pair coherence analysis was conducted with an alpha threshold of 0.05. Judgement of order coherence analysis was conducted with an alpha threshold of 0.008.

Next, we found that date-coherent judgements of order were associated with a greater confidence that date-noncoherent judgements of order, *t*(47) = 9.44, *p* < 0.001, *d* = 1.36, 90% IC [1.04, 1.71]. Again, participants were more confident in their answers that were in-line with their life story chronology than answers that conflicted with it (Table 1).

We found a main effect of task difficulty on confidence (Table 2). That is, intraperiod judgements of order were associated with a lower confidence than interperiod judgements of order, *t*(46) = 3.709, *p* < 0.001, *d* = 0.54, 90% IC [0.28, 0.80]. Participants were more confident in judgements of order that are supposingly easy compared to judgements of order that are supposed to be more difficult.

**Table 2:** Effect of task difficulty on the mean percentage of coherent pairs of memories and the mean percentage of date-coherent judgements of order, and on mean confidence.

	Interperiod (SD)	Intraperiod (SD)	Cohen’s d (90% IC)	p
Percentage of coherent pairs of memories	72 (17)	68 (18)	0.23 ([− 0.01, 0.47])	0.058
Percentage of date-coherent judgement of order	80 (15)	75 (14)	0.34 ([0.10, 0.59])	> 0.008
Confidence	6.17 (0.83)	5.92 (0.93)	0.54 ([0.28, 0.80])	< 0.001

Standard deviations are given in brackets. A coherent pair of memories was a pair in which the judgements of order were consistent across blocks (e.g. if a participant indicated first that their graduation occurred before their wedding, and second that their wedding occurred after their graduation). A noncoherent pair was a pair in which the two judgements were contradictory across blocks (e.g. if a participant indicated first that their graduation occurred before their wedding, and second that their graduation occurred after their wedding). A judgement of order was categorised as coherent or noncoherent with the chronology given by the dates (e.g. if the participant indicated that their graduation, 2012, occurred before their wedding, 2014, the judgement was coherent). Task difficulty was influenced by using interperiod pairs of memories (memories from two distinct lifetime periods) and intraperiod pairs of memories (memories from the same lifetime period). For these analyses, the alpha threshold was 0.008.

Finally, we designed a linear mixed model to assess a potential link between confidence and response time. We used confidence as a fixed effect and response time as a measure. Random effects relevance was tested using Bates et al.^36^ procedure. The test of the fixed effect of confidence on response time was significant, *b* = − 34.72, *t*(2357.548) =  − 5.39, *p* < 0.001. Thus, when judgement of order confidence was raised by one, response time decreased by 34.72 ms (lme4 R package,version 1.1–28^37^; *p*-values were calculated using Satterthwhaite approximation, available in the lmertest R package, version 3.1–3^38^). Thus, as frequently demonstrated, higher confidence was associated with a faster response time on the first-order task.

### Performance analysis

We did not replicate Skowronski et al.’s^30^ effects showing that judgements of order associated with memories from distinct lifetime periods were easier than judgements of order associated with memories from the same lifetime period (Table 1). The percentage of coherent pairs, that is the percentage of memory pairs associated with consistent judgement of order across blocks, did not differ when pairs contained memories from the same lifetime period, or memories from different lifetime periods, *t*(48) = 1.59, *p* = 0.058, *d* = 0.23, 90% IC [− 0.01,0.47]. The percentage of date-coherent judgements of order carried out with events from the same lifetime period did not differ from the percentage of date-noncoherent judgements of order carried out with events from different lifetime periods.

We analysed the effect of task difficulty on participants’ response time using a Wilcoxon signed-rank test, since the assumption of normality was not respected. This test showed a significant effect of task difficulty on response time, *V* = 167, *p* < 0.001, *r*_*c*_ = − 0.75, 90% IC [− 0.92, − 0.54]. Thus, intraperiod judgements of order were associated with faster median response times compared to interperiod judgements of order (respectively, *Mdn* = 1425 ms, *MAD* = 336.9; *Mdn* = 1638 ms, *MAD* = 317.2). A similar result was found with a parametric *t* test and the means, *t*(48) =  − 8.817, *p* < 0.001, *d* = − 1.26, 90% IC [− 1.59, − 0.95] (*M*_*inter*_ = 1438.4 ms, *SD* = 314; *M*_*intra*_ = 1693 ms, *SD* = 321.7).

## Discussion

Many control processes have been theorised when retrieving memories from the personal past but most of them are supposed to be nonconscious^6^. The aim of this study was to investigate the extent to which we can consciously reflect on the outputs of AM retrieval. To achieve this goal, we designed a judgement of order task that allowed us to assess awareness of the subjective organisation of AM. This task was accompanied by metacognitive ratings. We were interested in whether participants managed to distinguish answers that conflicted with their AM subjective organisation, and answers that were in-line with it.

Participants had a greater confidence in their coherent responses than in their noncoherent responses. That is, pairs of memories associated with two similar judgements of order, illustrating life story chronology, were associated with a greater confidence than pairs of memories associated with conflicting judgements of order. Furthermore, judgements of order which are in-line with the dates given by participants were associated with a greater confidence than judgements of order that contradicted these dates. These two results highlight the ability of participants to distinguish their right and wrong answers according to their autobiographical memory organisation. That is, participants showed metacognitive abilities in an autobiographical memory task. These findings contradict results from Burt et al.’s^24^ study showing no links between performance and confidence in a similar task. Burt et al.’s primary interest was to evaluate whether participants were able to recall the order of the events they experienced coherently with the actual chronology of the events. They compared AM organisation to the objective chronology and found differences between the two orders. Thus, their confidence judgements evaluated the ability to distinguish answers that were in line with the real chronology to answers that conflicted with it. They found no link between confidence and performance, suggesting that their participants did not know whether they were recalling an order coherent with the diary or when they were recalling an incorrect order. In the current experiment, we assumed that participants judged whether their answers conflicted or not with the subjective chronology of their AM, based on the idea that consistent answers reflected AM organisation. Our metacognitive measures assessed monitoring of a coherent subjective representation of the personal past whereas Burt et al. measured the ability to distinguish differences between AM and the real world. Taken together we may postulate that metacognitive monitoring is more accurate for subjective organisation (coherence) than it is for the veracity of our recall (correspondence) according to Goldsmith and Koriat’s^39^ conceptualisation.

The metacognitive performance observed in this AM task presents similar properties to function assessed in different types of memory tasks (for a review see Ref.^40^). Confidence was not only related to participants’ first order performance, but also by participants’ first order reaction time. We found that faster judgements of order were associated with higher confidence^20^. Furthermore, although we did not find an effect of task difficulty on performance, interperiod judgements of order, which we might imagine were experienced as easier despite our lack of an effect for this manipulation, were associated with a greater confidence than intraperiod judgements of order^29, 30^. As well as numerous methodological differences, the failure to replicate Skowronski et al.’s effect showing a difference of performance between intra and interperiod judgements of order could possibly be due to the fact that our task was harder than theirs’ (i.e. overall, participants had 70% (SD = 17) pairs of memories associated with two coherent judgements, and 77% (SD = 15) date-coherent judgements against a mean of 84% of date-coherent judgements in Skowronski et al.). The difference in confidence could be related to the difference of response time observed between interperiod and intraperiod judgements of order that we found. The fact that interperiod judgements of order were performed faster than intraperiod judgements could have led to a greater confidence. This difference in fluency could have modified the subjective difficulty of the task^41^, even if difficulty did not influence participants’ performance. Thus, interperiod judgements of order could have felt easier than the intraperiod judgements of order.

The present study adds to data examining metacognitive confidence across domains, since to our knowledge, this is the first task to operationalise a measure of confidence in a yes/no decision for autobiographical materials. As such, whilst we cannot speak to the individual differences in confidence across domains, we have at least shown that, as hypothesised in the domain general account of metacognition, we are indeed able to reliably ascribe confidence values to contents from autobiographical memory in an order judgement task^42, 43^. Future research could consider whether, as is the case for retrospective confidence judgements across domains (e.g. episodic and semantic memory; see Ref.^17^), people who are more accurate at evaluating their confidence in autobiographical memory retrieval are also more accurate on other memory retrieval tasks.

This study proposed a new design to assess metacognitive abilities in a task taking into consideration the subjective nature of autobiographical memory. However, the fact that our main hypothesis was based on the mean of two judgements (i.e. each pair of memories was associated with the mean of confidence of its two related judgments of order) raises a possible limitation. By using average confidence scores, we lost information about confidence directly associated with right and wrong answers (although note that when we looked at the responses according to the dates provided by participants we still found the same pattern of results). Future research could compare confidence associated with judgements of order in line with life story chronology and confidence associated with judgements of order that conflicted with this chronology by establishing more concretely which judgement differs from the established chronology.

One possibility is that our findings are contributed to by the ability to order information more generally, rather than specifically AM events, especially since across blocks there was a switching component. We asked which of two events occurred first in time, once in one direction and then in the other. Our hypothesis was that naturally, people with a less developed idea of autobiographical chronology would provide more incoherent responses across the two blocks (‘occurred before’ and ‘occurred after’) than those people with a well developed lifespan chronology. It is possible, however, that people struggled with the ordering task per se. It is also possible that rather than drawing on autobiographical memory directly, participants remembered their previous response on the task, and this produced the results here. It would be therefore interesting to test the validity of ordering judgements alongside more traditional measures of autobiographical accuracy (with the expectation that our AM ordering task is related to performance on typical AM tasks). Also, it could be of interest to compare metacognition for our AM ordering tasks with an ordering task using public dates, or simple orders such as the months of the year.

To conclude, the present study showed that participants were able to evaluate their performance in an autobiographical memory task. More precisely we showed that they managed to distinguish judgements of order that conflicted with their life story chronology from judgements of order that were in line with this chronology. Even if we failed to replicate results from previous study^29, 30^, we found that metacognitive abilities observed in this task shared similar properties with metacognitive abilities assessed in other memory tasks. That is, first order fluency and the sensation of difficulty could have produced the pattern of findings here^20, 41^. Whereas more sophisticated tasks will need to be developed to address metacognitive control processes online in autobiographical memory, it remains the case that people can at least accurately ascribe confidence in line with the coherence of the judgements of order from events from their personal past. It would be of interest to see whether this pattern is maintained in populations proposed to have autobiographical memory retrieval which is impoverished or disorganised^44, 45^. The findings are of importance for models of autobiographical retrieval, since it is a first step in supporting the claim that metacognitive processes operate in retrieval in AM. It would be of interest next to consider how such evaluations relate to decisions to report or withhold AM information (e.g. Ref.^46^), or in grain-size reporting (e.g. Ref.^47^).

## Method

### Participants

An a priori power analysis was conducted for a unilateral one-sample t-test with an alpha threshold of 0.05, a power of 0.80 and a medium Cohen’s *d* of 0.30 using the pwr R package (version 1.3–0^48^). This estimated a required sample size of 71 participants. In order to get this number of participants after the application of our exclusion criteria, we increased this estimation by 15% thus we aimed to recruit 82 participants.

Eighty-three participants (*M*_age_ = 25, *SD*_age_ = 8.6, 72% of females, 61% of French speakers) volunteered for this experiment. Participants who failed at least one attentional check, participants who did not write appropriate memories (e.g. a participant wrote *rien*, (“nothing”), for the majority of their memories) and those without data (e.g. because of technical issues) were excluded. Finally, data from 51 participants were analysed (*M*_age_ = 24.2, *SD*_age_ = 6.9, 70% of females, 78% of French speakers).

Participants were recruited through social media. Participants had to be over 18 years old, they had to be (or have been) university student (or equivalent) and they had to carry out the experiment on a computer. The experiment was available both in French and English. On average, the experiment lasted approximately 20 min. Participation was on a voluntary basis and no compensation was offered. This study was not approved by a named institutional and / or licensing committee. In France, researchers do not seek ethics approval when research involving humans are not concerned by the Law No. 2012-300 of March 5, 2012 of the Public Health Code. However, we followed the Code of Ethics of the World Medical Association (Declaration of Helsinki) for experiments involving humans and participants gave their informed written consent before the experiment. Also, we followed the ethical recommendations of the American Psychological Association (APA).

### Materials and design

This experiment was implemented on Psychopy (version 2021.2.3^33^) with Python language (version 3.6.6^49^) and was exported online on the website Pavlovia (https://pavlovia.org/^33^) by using Javascript language (version ECMAScript 2021^50^). Each participant generated 12 memories at the beginning of the experiment. Half of the memories were memories from their high school, and the other half of the memories were memories from the time they were at university (or equivalent). Each memory had to be summarised with three or four words. It had to be related to a unique event, and not to a repeated one, and to an event that did not last longer than 1 day. Four memories from each period were randomly selected. After that, participants’ memories were grouped into pairs. All non-repetitive pairs of memories were created, thus 28 pairs were created. Pairs containing memories from the same lifetime period were categorised as intraperiod pairs and pairs containing memories from different lifetime periods were categorised as interperiod pairs.

### Procedure

At the beginning of the experiment, participants gave their consent and their demographic details. Next, they had to generate 12 memories. First, participants had to generate memories as described above. After the memory generation participants were presented instructions for the judgement of order task.

Participants carried out two blocks of judgement of order. Each block had two parts: a training part followed by an experimental part. The training part used pairs of well-known public events adapted to the French and English-speaking populations, and the experimental part was conducted with the pairs of memories generated by participants. The training phase ended with an easy judgement (i.e. judgement of order for World War I and World War II) that served as an attentional check. Participants completed two counterbalanced blocks (i.e. training phase, attentional check, experimental phase), one block corresponding to each temporal direction in the judgements. In one block, they had to indicate which event of every pair occurred first (earlier), in the other they had to indicate which event of every pair occurred last (later). The order of the blocks was random. At the end of the experiment, participants also indicated the date of occurrence of every memory they generated.

**Figure 2:**
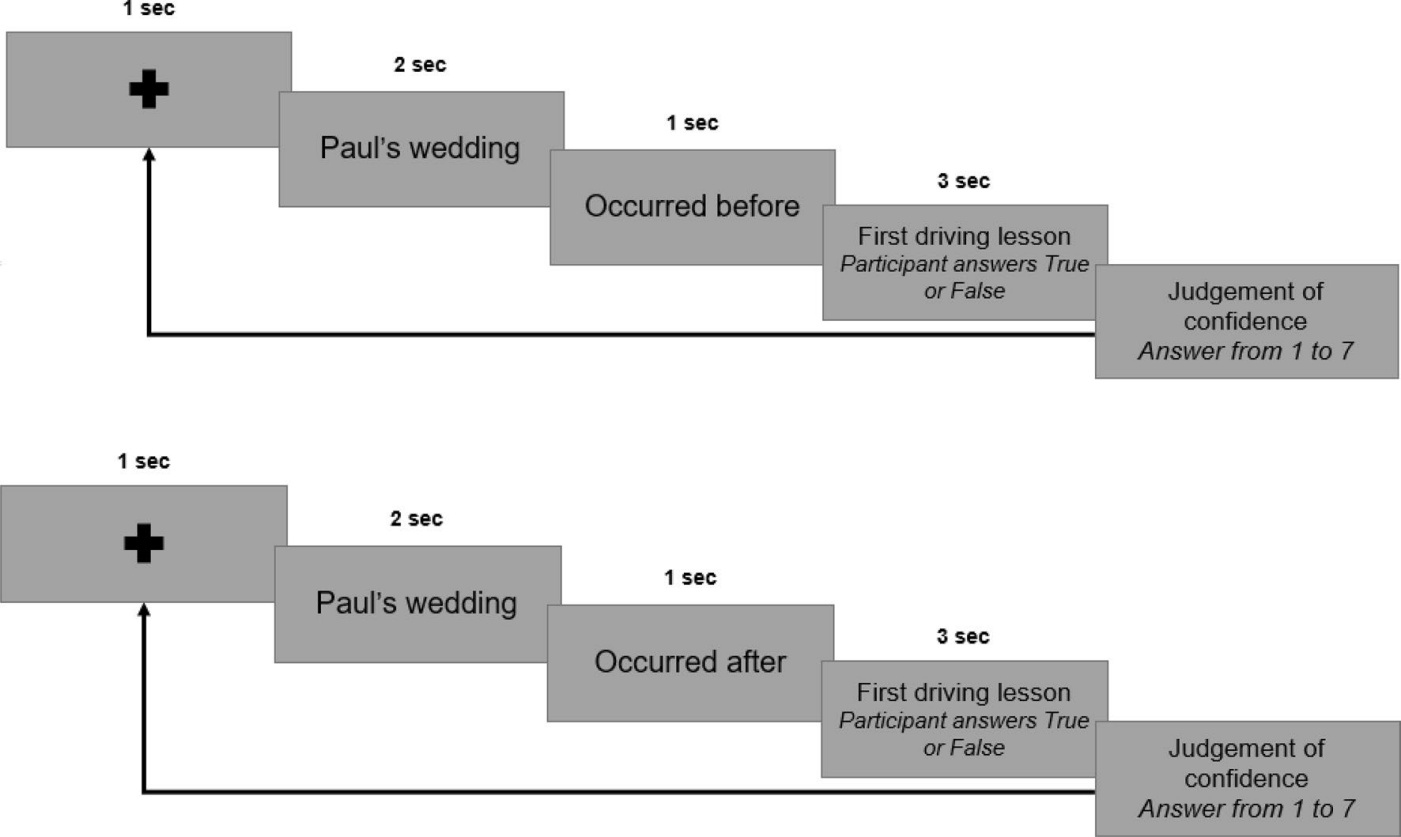
Procedure used during order judgements. In two counterbalanced blocks participants had to judge if a target memory occurred before (upper panel) or after (lower panel) a second target memory. Each block contained 28 judgements of order with memories.

Every judgement of order followed the same procedure (Fig. 2). The first event of a pair was presented, followed by an affirmation. The affirmation was either “Occurred before” or “Occurred after”. Then the second memory of the pair was presented. Participants made a judgement as to whether the affirmation was true (e.g. if the first presented event occurred before the second one) or false (e.g. if the first presented event did not occur before the second one). They pressed the “V” key of their keyboard to indicate that the affirmation was true, and the “F” key if the affirmation was false. They were instructed to answer as accurately and as rapidly as possible. They were given 3 s to answer, otherwise no answer was recorded. After their answer, they indicated their confidence on a 1–7 scale with seven being the highest level of confidence.

## Data Availability

Scripts (R, Python, Javascript), auxiliary results, and data can be found on OSF: https://osf.io/wzt2p/?view_only=544050b3f69948d089ed405932299e3f.
